# Design of a soft sensing technique for measuring pitch and yaw angular positions for a Twin Rotor MIMO System

**DOI:** 10.12688/f1000research.51894.1

**Published:** 2021-05-05

**Authors:** Sneha Nayak, Sravani Vemulapalli, Santhosh Krishnan Venkata, Meghana Shankar

**Affiliations:** 1 Department of Instrumentation and Control Engineering, Manipal Institute of Technology, Manipal Academy of Higher Education, Manipal, Karnataka, 576104, India

**Keywords:** Kalman filter, Twin Rotor MIMO system, analytical redundancy, neural network, Luenberger observer, soft sensing

## Abstract

Background: This paper presents a soft sensor design technique for estimation of pitch and yaw angular positions of a Twin Rotor MIMO System (TRMS). The objective of the proposed work was to calculate the value of pitch and yaw angular positions using a stochastic estimation technique.

Methods: Measurements from optical sensors were used to measure fan blade rotations per minute (RPM).  The Kalman filter, which is a stochastic estimator, was used in the proposed system and its results were compared with those of the Luenberger observer and neural network. The Twin Rotor MIMO System is a nonlinear system with significant cross coupling between its rotors.

Results: The estimators were designed for the decoupled system and were applied in real life to the coupled TRMS. The convergence of estimation to the actual values was checked on a practical setup. The Kalman filter estimators were evaluated for various inputs and disturbances, and the results were corroborated in real time.

Conclusion:  From the proposed work it was seen that the Kalman filter had at least Integral Absolute Error (IAE), Integral Square Error (ISE), Integral Time Absolute Error (ITAE) as compared to the neural network and the Luenberger based observer.

## Introduction

**Figure 1.**
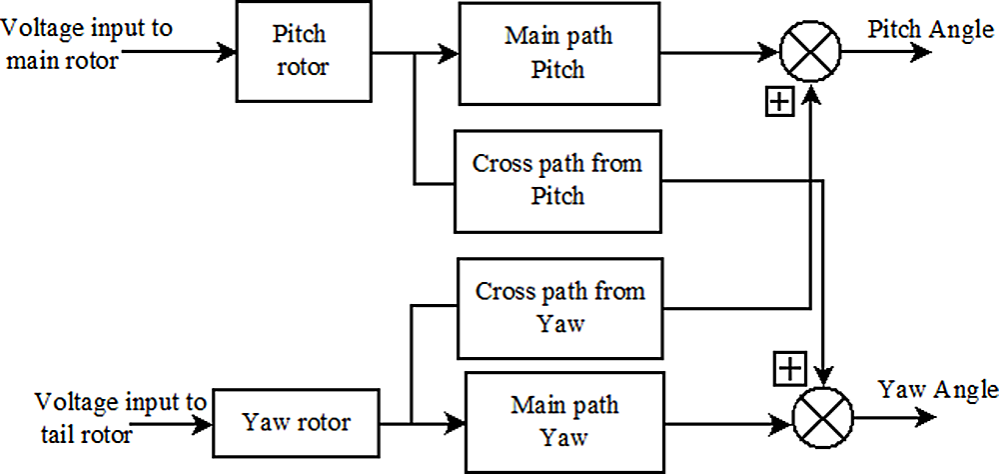
Block diagram of basic twin rotor multi input multi output system.

Study of aerospace systems has always been a subject of interest by many researchers, engineers, and technical students. However, it is practically very difficult to analyse these aerospace systems, so a replica is designed to understand the behaviour of the actual systems. One such system, which replicates the behaviour of a helicopter system, is a TRMS. The TRMS has two of the three movements of a helicopter, pitch angle and yaw angle. ^1^ The TRMS position is controlled by the rotor speed. The two input variables to the TRMS are voltage to the main rotor and tail rotor, and the outputs are the pitch and yaw angle as shown in Figure 1. Excitation to these motors is given by a controller based on a set point given by user. Desired control action can be achieved only by accurate measurements of pitch and yaw.

Several researchers have reported work on computing the yaw and pitch positions using different techniques. ^1– 5^ Different algorithms are incorporated to maintain and retain the stability of the TRMS. Controllers like Proportional Integral Derivative (PID), fuzzy PID, sliding mode controller, fuzzy sliding mode controllers and estimators like Luenberger and Kalman are incorporated on the TRMS. ^2^ Rahideh and Shaheed ^3^ discussed the design of a model predictive controller for a TRMS. The controller is simulated based on the state model using motor armature current and angular speed as inputs. Jahed and Farrokhi ^4^ discussed the design of a fuzzy based robust control for a TRMS using angular speed given by tachometer in a simulation platform, and Tao *et al.* ^5^ discussed the fuzzy sliding and integral sliding controller design for a TRMS. In Rohith, ^6^ a new control law was proposed for the design of sliding motor controllers, which mitigate the chattering problem with the gain variation and thereby guarantees faster system response and robustness. Design of an auto tuning based PID controller with fractional-order reference model approximation for a DC rotor in a TRMS model is discussed in Alagoz *et al.* ^7^ The data regarding the process variable is derived from the angular position of pitch and yaw. In Patel and Janardhanan, ^8^ a tuning method based on the Moore Skelboe Algorithm for a PID controller was presented. This was effective in finding the optimal PID values for the given initial range and applicable for some of the higher-order Linear Time Invariant (LTI) systems as well as for all first and second order linear time invariant systems. Further the method was effective in stabilizing unstable systems. Halim and Ismail ^9^ presented a PID controller tuning using tree physiology optimization, which was based on the tree growth concept whose simulation results showed better results compared to other tuning methods in Single Input Single Output (SISO) and MIMO problems. Rao *et al.*, ^10^ reported design of an observer using robust PID controller logic with *H*∞ observer to obtain the stable output in TRMS with sensor and actuator failure. Netto *et al.*, ^11^ reported an Adaptive PID controller to cancel the effect of cross coupling between the tail rotor and main rotor when operating simultaneously in a TRMS. Adaptive linear quadratic regulator design for stable system operating at a single reference point with six tail and main rotors is reported by Faisal and Omar Waleed. ^12^ Ghellab *et al.*, ^13^ reported an adaptive radial basis function neural network with a dynamic terminal sliding mode control with cross coupling between tail and main subsystem for tracking the set point in the presence of wind gust and other external disturbance.

In Sleimi *et al.* ^14^ a linear time-varying controller was designed using a differential flatness property leading to a two Degree of Freedom (DOF) controller for which the system must be in canonical controllable form with no need to define its dynamics. In Panda *et al.* ^15^ the proposed control strategy used an adaptive back stepping controller implemented on a Twin Rotor Multi Input Multi Output System. It provided an explicit relationship between the saturation bound of the input signal and upper bounds of tracking errors, uncertainties, and disturbances. Mondal and Dey ^16^ presented the development of a two DOF control system design providing an additional degree of freedom depending on the nature of the plant and loop compensators. The design methodology can be implemented for integer as well as non-integer order plants with better tracking and loop robustness.

Neural network based controller design using model inversion control for a twin rotor MIMO system was reported in Rahideh *et al.* ^17^ Design of a differential evolution based neural network model to control the TRMS with data of angular positions was reported by Subudhi and Jena. ^18^ Pratap and Purwar, ^19^ reported implementation of a neuro-adaptive robust back stepping controller for TRMS. Many researchers have worked on the TRMS, but the design technique of most controllers is based on the data received from the sensor. If the sensor data is erroneous the complete system fails. To overcome the above problem, this paper attempts to design a soft sensor or observer for computing the actual yaw and pitch data to the controller. In order to start the design of a soft sensor it is essential to understand the TRMS model.

The TRMS dynamics are given in Rahideh *et al.*, ^20^ Sun and Song ^21^ and Ahmad *et al.* ^22^ It was found that the system was a nonlinear and coupled one with considerable error in the measurements. The method based on the Kalman filter is considered as it provides for updating the estimates based on errors, and also takes into account the process and the measurement noise uncertainties to give out the best estimate. Model dynamics of the system are known and are used for estimation. The Kalman filter is widely used as a state estimator and is used in various fields. For example, the Kalman filter is used to estimate track soil parameters, ^23^ for tracking objects in videos based on color features and mean shift, ^24^ for estimation of flow rate and isolation of occurrence of fault, ^25^ for estimation of wind speed and comparison with the estimation from Takagi-Sugeno observers. ^26^

**Figure 2.**
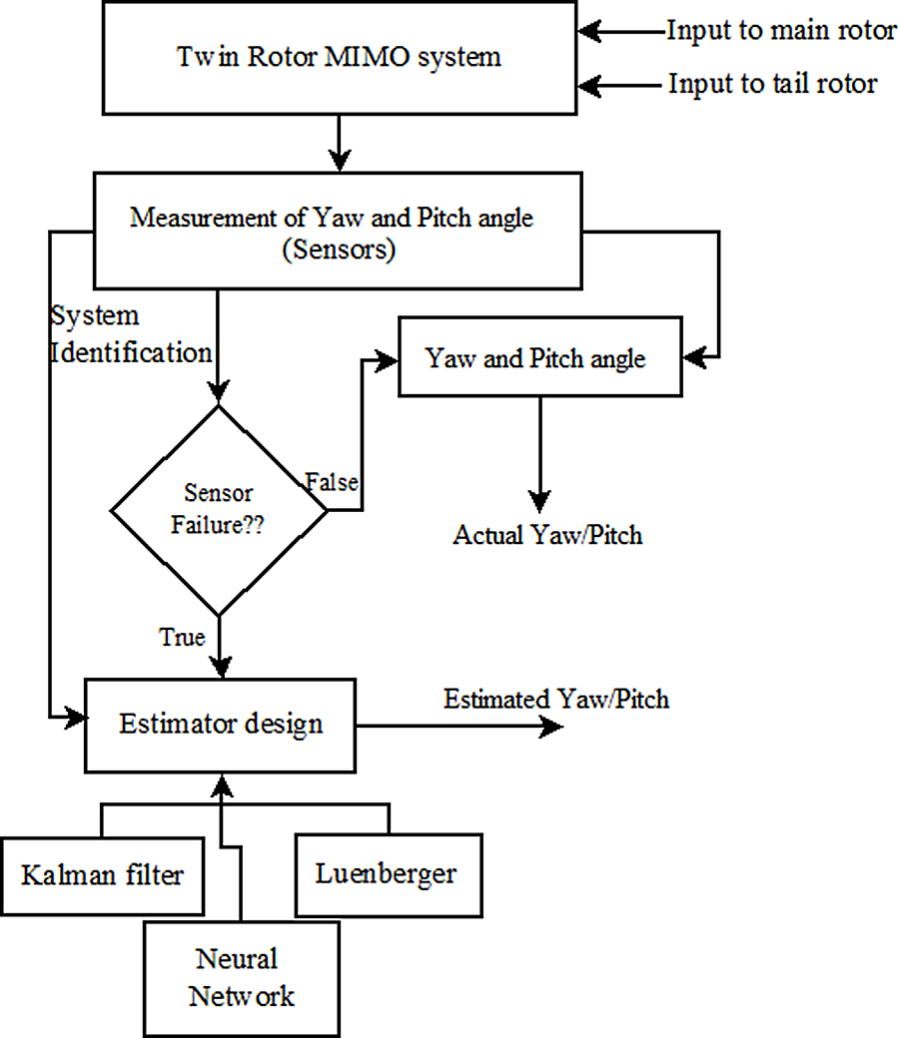
Outline of the proposed work.

The TRMS is a prototype device used to understand the dynamics of a helicopter system. It consists of two rotor fans to operate the device in two Degrees of Freedom (2-DoF). For controlling/stabilizing the system performance based on a desired set point, it is essential to sense the actual pitch and yaw positions. The presence of faults during data transmission to the controller lead to the controller failing to take the desired action and thus the system destabilizes. Hence, a technique is proposed with the objective of designing an estimator so that it can be used in case of any contingencies. The Kalman filter technique is used to design the estimators for estimating the pitch and yaw position of the TRMS. In order to verify the effectiveness of the algorithm, it is applied to the system in real life and compared with the actual trajectory of the response. The performance of the designed Kalman filter for estimating angular pitch and yaw information is compared with the Luenberger observer and neural network estimator to evaluate its performance. The outline of the work carried out is shown in Figure 2.

## Methods

### Experimental setup

**Figure 3.**
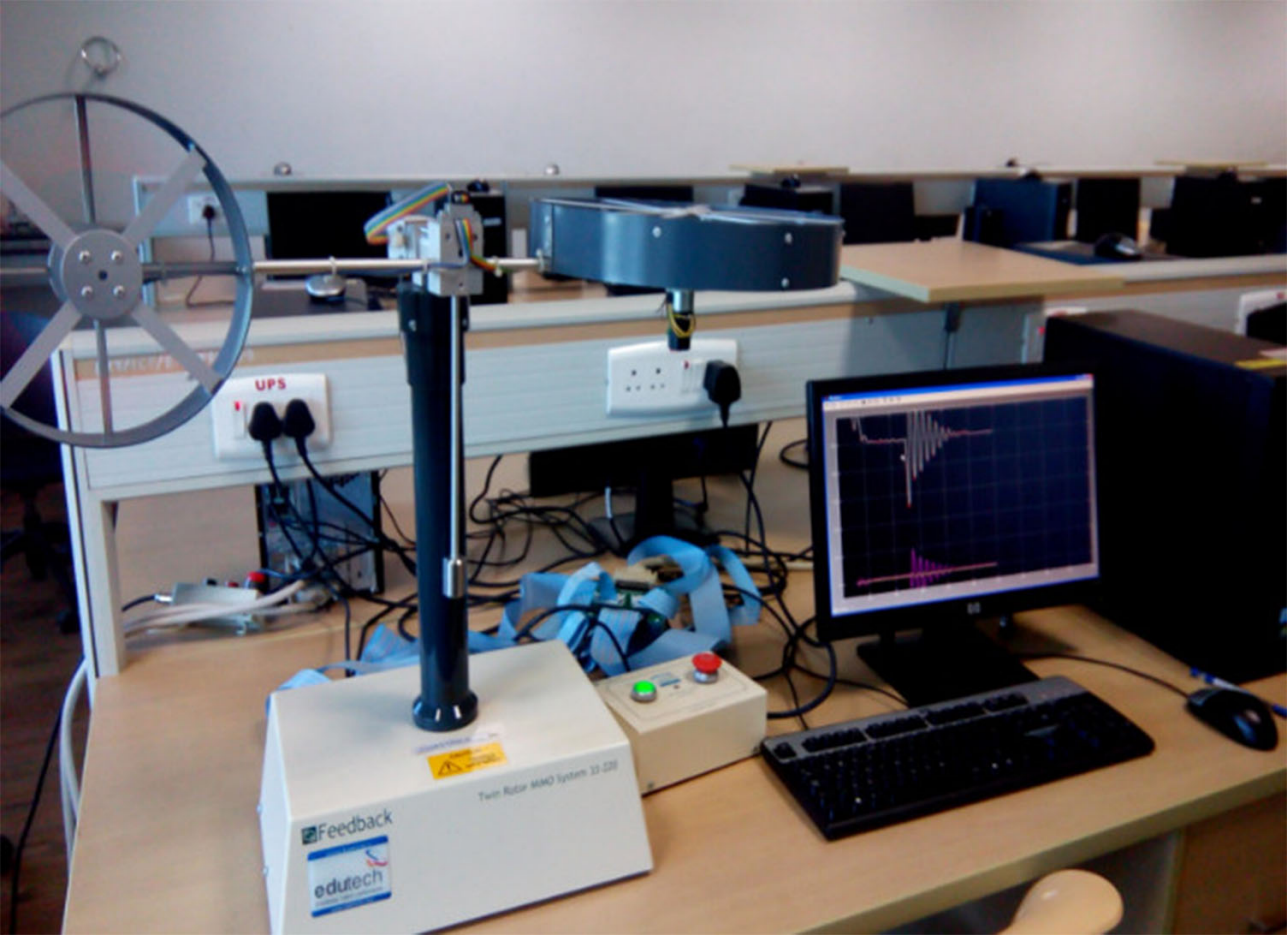
Experimental setup used to demonstrate the proposed work.

The twin rotor MIMO system is a prototype of a helicopter propeller system. It is shown in Figure 3. The system was a non-linear MIMO system with significant cross-coupling. The angle of attack of the rotors was fixed and the aerodynamic forces were controlled by varying the speed of the motors. Significant cross-coupling was observed between the actions of the rotors, with each rotor influencing both angle positions. Two propellers were driven by DC motors controlled by their supply voltages. The measured signals were positioned on the beam in the space i.e. two position angles which are measured by rotary optical encoders mounted on each of the rotor shafts. The measured output was obtained from the rotary optical encoders. It was calibrated for the inertial axis using the loop up table approach. ^19, 20^

### Estimator design

**Figure 4.**
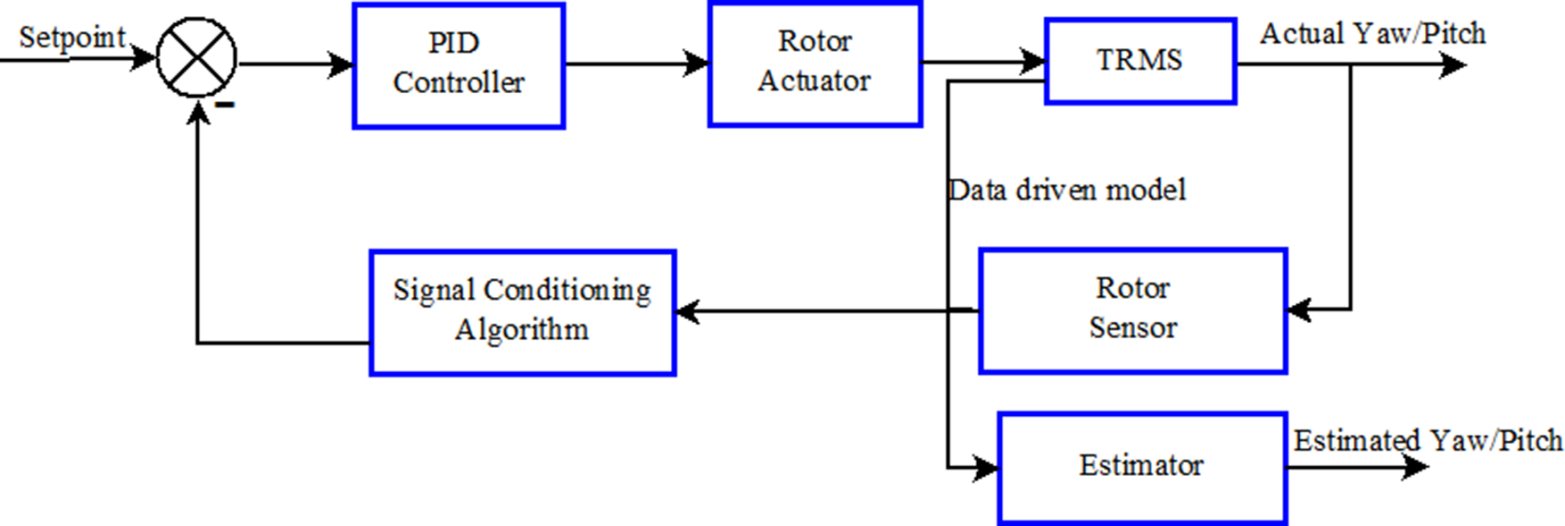
Block diagram of the closed loop Twin Rotor multi input multi output system.

The TRMS has two modes of operation, namely, 1-DOF control and 2-DOF control. In 1-DOF control, the pitch and the yaw are controlled individually and independently, whereas in 2-DOF control the pitch and the yaw are controlled simultaneously in a coupled system. The block diagram of the proposed scheme is shown in Figure 4. Here the coupled TRMS was controlled by two separate PID controllers for the pitch and the yaw respectively. The controllers were tuned to maintain the angles at the desired positions or set point, except in the event of some contingency such that the pitch or the yaw failed to maintain the desired position. The transient output of the sensors was used to design an estimator to find the angles where models were generated using a system identification process. A decoupled pitch and yaw model was used for designing the estimator. In this case pitch and yaw angles were independently calculated using the Kalman filter.

### Kalman filter

The Kalman filter is developed from the Bayesian filter. It was initially used to extract the signal from the noisy output data of sensors, and/or actuators. ^27– 29^ Over time, it has been used as a state estimator also. It is a stochastic estimator and requires explicit modeling of the process noise and the measurement noise in addition to the system model. The Kalman filter estimation is a two-step process. Initially it generates an estimate from the knowledge of the system dynamics, which is embedded in the system model along with the noise model. This is called the apriori estimate. Once the measurement output is available, the apriori estimate is updated to the posteriori estimate. The second stage involves the Kalman gain which is altered in every step based on an optimization problem. The Kalman gain was the main feature of this estimator and it decided if the estimate derived much of its information from the measurement output or the system dynamics. Hence, the Kalman gain was also updated on every step such that the error between the actual output and the estimate was reduced i.e. the residue reduced to zero. The noise covariance matrix was also updated at each step based on the error. Here the Markovian model was used where the state was said to be Markovian but the measurement was usually not Markovian. Also, we considered the process noise and measurement noise as Gaussian and they were not correlated with each other. It is also called the Gauss-Markov model. This allows nonlinearity. The mean was assumed to be zero for both measurement and process noises. Covariance was chosen in this case such that the covariance of measurement noise was slightly less than the covariance of the process noise. In general, the probability distribution of the state is given by equation (1)

$$p\left(X_{k+1}\right)=\int p\left(X_{k+1}|X_{k}\right)p\left(X_{k}\right)dX_{k}$$ (1)

where $p\left(X_{k+1}\right)$ was determined by $p\left(W_{k}\right)$ which was the probability distribution of the process noise. The system state $X_{k}$ was modeled as a linear combination of the previous states along with the input U and the process noise W, as given in equation (2)

(2) $$X_{k}=FX_{k-1}+GU_{k}+W_{k-1}$$

For the considered TRMS, the matrices for the decoupled pitch model were found to be:

(3) $$\begin{array}{l} F_{y}=\left(\begin{array}{ccc} -1.38 & -1.6456 & -14.7611\\ 0.9244 & -2.5724 & -31.1124\\ -0.0196 & 0.3346 & -8.0476 \end{array}\right);\;G_{y}=\left(\begin{array}{c} \begin{array}{c} 1\\ 0 \end{array}\\ 0 \end{array}\right)\\ S_{y}=\left(0.0010.03360.4065\right);\;D=0 \end{array}$$

The matrices for the decoupled yaw model were found to be:

(4) $$\begin{array}{l} F_{y}=\left(\begin{array}{ccc} -1.38 & -1.6456 & -14.7611\\ 0.9244 & -2.5724 & -31.1124\\ -0.0196 & 0.3346 & -8.0476 \end{array}\right);\;G_{y}=\left(\begin{array}{c} \begin{array}{c} 1\\ 0 \end{array}\\ 0 \end{array}\right)\\ S_{y}=\left(0.0010.03360.4065\right);D=0 \end{array}$$

The measurement obtained was modeled as a linear combination of the system states and measurement noise V as in equation (5)

(5) $$Z_{k}=HX_{k}+V_{k}$$

In the TRMS model, the measured output i.e. pitch and yaw, were related to states directly through output matrices S i.e. $H_{p}=S_{p}$ , $H_{y}=S_{y}$ . Hence for pitch, $H_{p}=\left(0.0166\;0.4194\;2.454\right)$ . For Yaw, $\;H_{y}=\left(0.001\;0.0336\;0.4065\right)$ . Let Q and R be processed noise covariance and measurement noise covariance respectively. The apriori error covariance P is given by equation (6).

(6) $$P_{k}=FP_{k-1}F^{T}+Q$$

Once the measurement was obtained, the Kalman gain was computed and the apriori state estimates and the apriori error covariance were updated as follows:

**Kalman gain**:

$$K_{k}=P_{k}H^{T}/\left(HP_{k}H^{T}+R\right)$$ (7)

The posteriori estimate:

$$\hat{X_{k}}=X_{k}+K_{k}.\left(Z_{k}-HX_{k}\right)\;$$ (8)

The posteriori error covariance

$$\hat{P_{k}}=\left(1-{\mathrm{HK}}_{k}\right).P_{k}$$ (9)

Here $\hat{X_{k}}$ was the estimate of the state at k given the measurement at k and the apriori estimate $X_{k}$ at time k. Also, it should be noted that the Kalman filter was a one step ahead predictor. The noise covariance matrices R and Q were the most complex matrices to compute, therefore the idea was to start with an initial estimate of identity matrices for both R and Q and to change them based on the convergence between the actual and the estimated outputs. The Kalman filter estimated the states. However, the objective was to estimate the pitch and the yaw which were the output of the TRMS. Hence, the output was obtained from equation (10).

$$Y=S.\hat{X_{k}}\;$$ (10)

and the output residue (error) was given by equation (11)

Error,

(11) $$e\left(t\right)=Y_{\text{residue}}=Y\prime \left(k\right)-Y\left(k\right)\;$$

where $Y^{\prime }\left(k\right)$ was the actual value of pitch and yaw

### Estimation with the Luenberger observer and neural network

The Kalman filter provided a stochastic method of estimation, whereas the Luenberger observer and neural network provided a deterministic method of estimation of the states. The estimation of yaw and pitch carried out with these deterministic methods had a poorer performance due to their inability to incorporate uncertainties that emerge inherently in any real systems, such as the uncertainties due to modeling errors or sensor output errors. However, these estimates can be feasible if the error margin is not very stringent.

**Figure 5.**
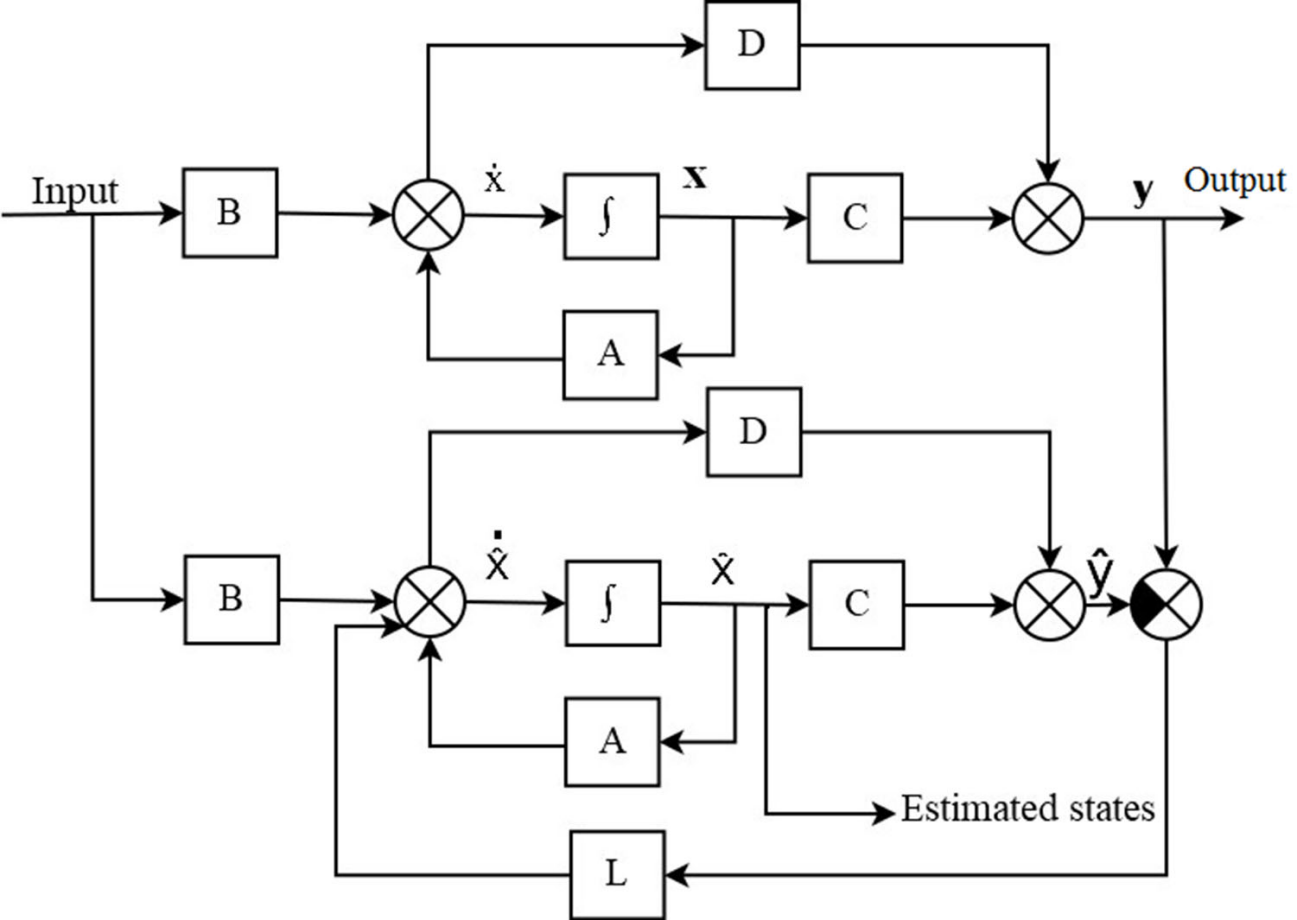
Block diagram of the Luenberger Observer used for estimation of pitch and yaw.

Luenberger based observers are widely used for numerous applications and modified according to the type of the system. ^27^ The extended Luenberger observer and adaptive Luenberger observer are the latest class of these observers. ^27^ The Luenberger observer is used for estimation of battery charge for electric vehicles, ^30^ flux in motor drives, ^31^ motorcycle dynamics, ^32^ sensor-less speed estimation. ^33^ The scheme of this observer is shown in Figure 5. The following were the expressions for the observer design from Figure 5.

Assume a plant:

$$\dot{x}=\mathrm{Ax}+\mathrm{Bu}$$ (12)

y=Cx (13)

State equations of the observer:

$$\dot{\hat{x}}=A\hat{x}+\mathrm{Bu}+L\left(y-\hat{y}\right)$$ (14)

$$\hat{y}=C\hat{x}$$ (15)

$$\dot{x}-\dot{\hat{x}}=\left(A-\mathrm{LC}\right)\left(x-\hat{x}\right)$$ (16)

$$\dot{e_{x}}=\left(A-\mathrm{LC}\right)e_{x}$$ (17)

$$y-\hat{y}=Ce_{x}$$ (18)

From equation 17, it is understood that if the eigenvalues were all negative, the estimated state vector error, e _x_, would decay to zero. The design then consisted of solving for the values of L to yield a desired characteristic equation.

$$\det\left[\mathrm{sI}-\left(A-\mathrm{LC}\right)\right]=0$$ (19)

Then the selection of eigenvalues for the observer was carried out to achieve a required closed loop response. These eigenvalues determined a characteristic equation that was made equal to equation 19 to solve for L. The poles for the observer for both pitch and yaw were chosen to be at -1, -2, and -3.

The Luenberger gain L for pitch was:

$$L=\left(\begin{array}{c} -2.9012\\ 11.8917\\ 2.2713 \end{array}\right)$$ (20)

The Luenberger gain L for yaw was:

$$L=\left(\begin{array}{c} 35.3716\\ 76.5373\\ 19.7974 \end{array}\right)$$ (21)

**Figure 6.**
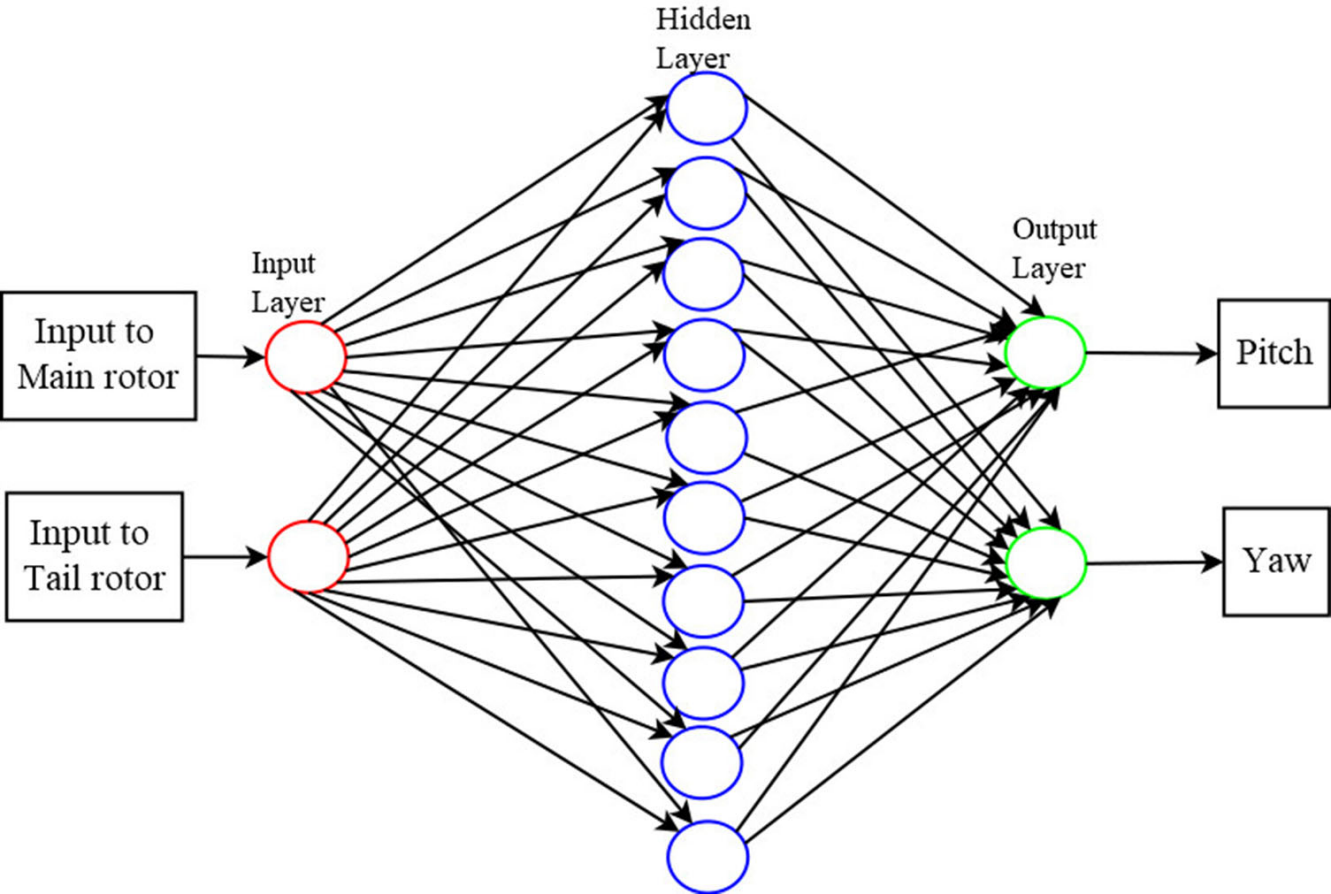
Neural network model used for estimation of pitch and yaw.

**Figure 7.**
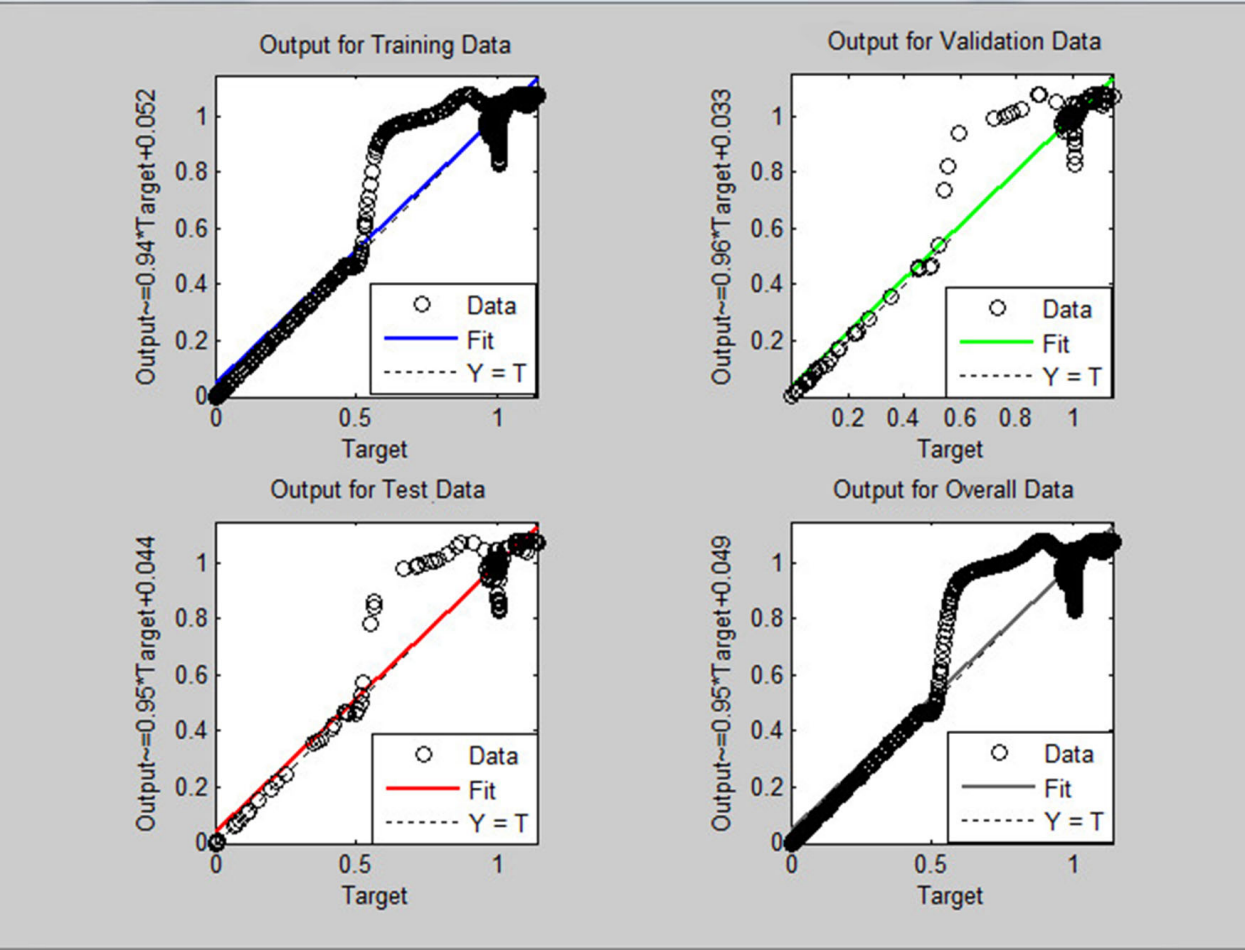
Neural network regression graph for the pitch system.

**Figure 8.**
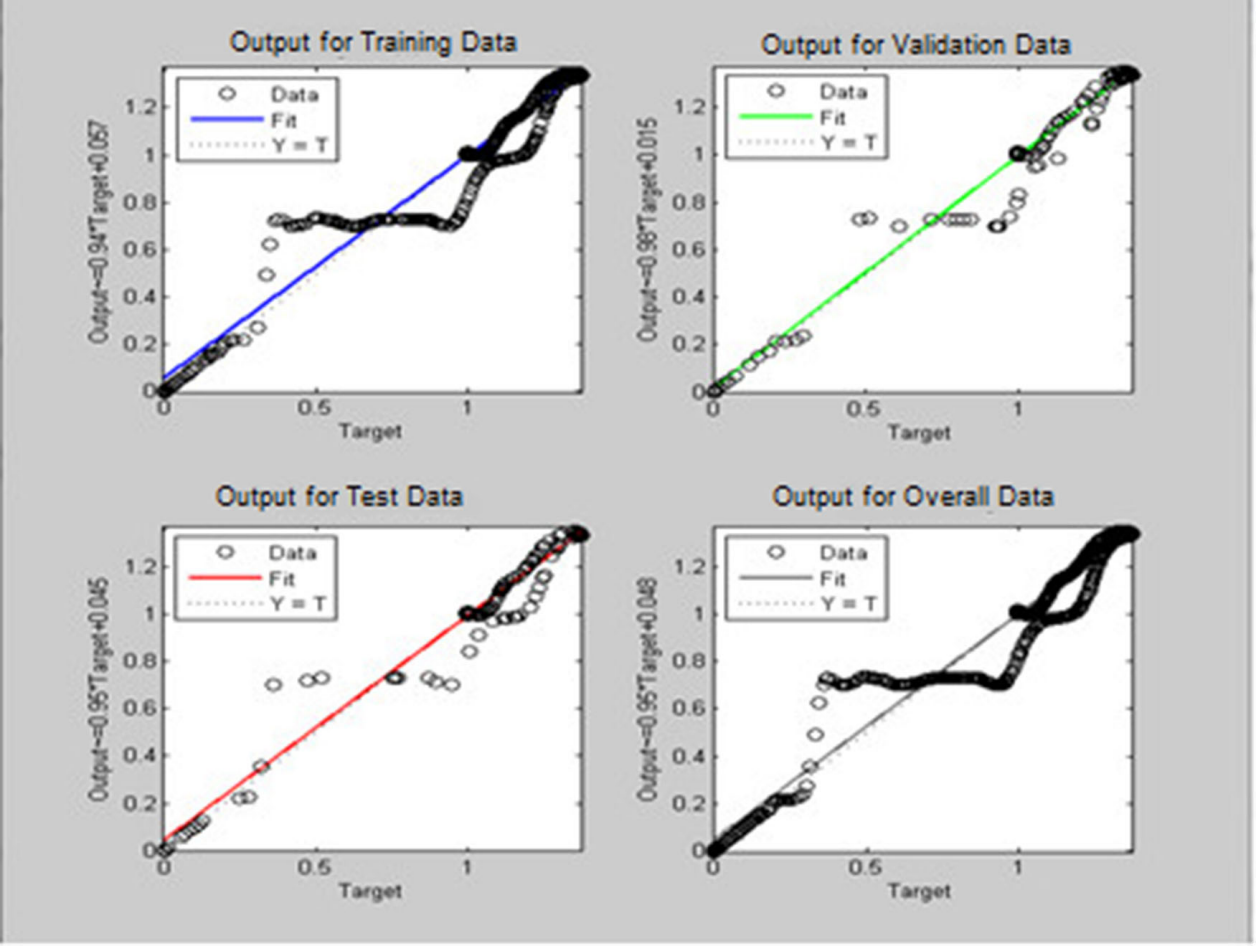
Neural network regression graph for the yaw system.

The estimation based on the neural network was based on a time series correlation between the input and the output, also called the targets. This assumed a black body model, where the dynamics of the system were not explicitly parameterized and the forecasting was done only from the input output relationship of the system. Here the neural network was trained to replicate the behavior of the system by using the given set of input output pair of data. The *Levenberg-Marquardt* algorithm with a back-propagation network having one hidden layer with a size of 10 neurons, which was used to predict the output i.e. the pitch and the yaw from the input. The neural network used for the present work is shown in Figure 6. The ‘nftool’ of MATLAB version R2014a, ^34^ provided the platform to train the neural network and this was used in the current work. The regression graph for the trained neural network is shown in Figure 7 for pitch and Figure 8 for yaw systems.

## Results

**Figure 9.**
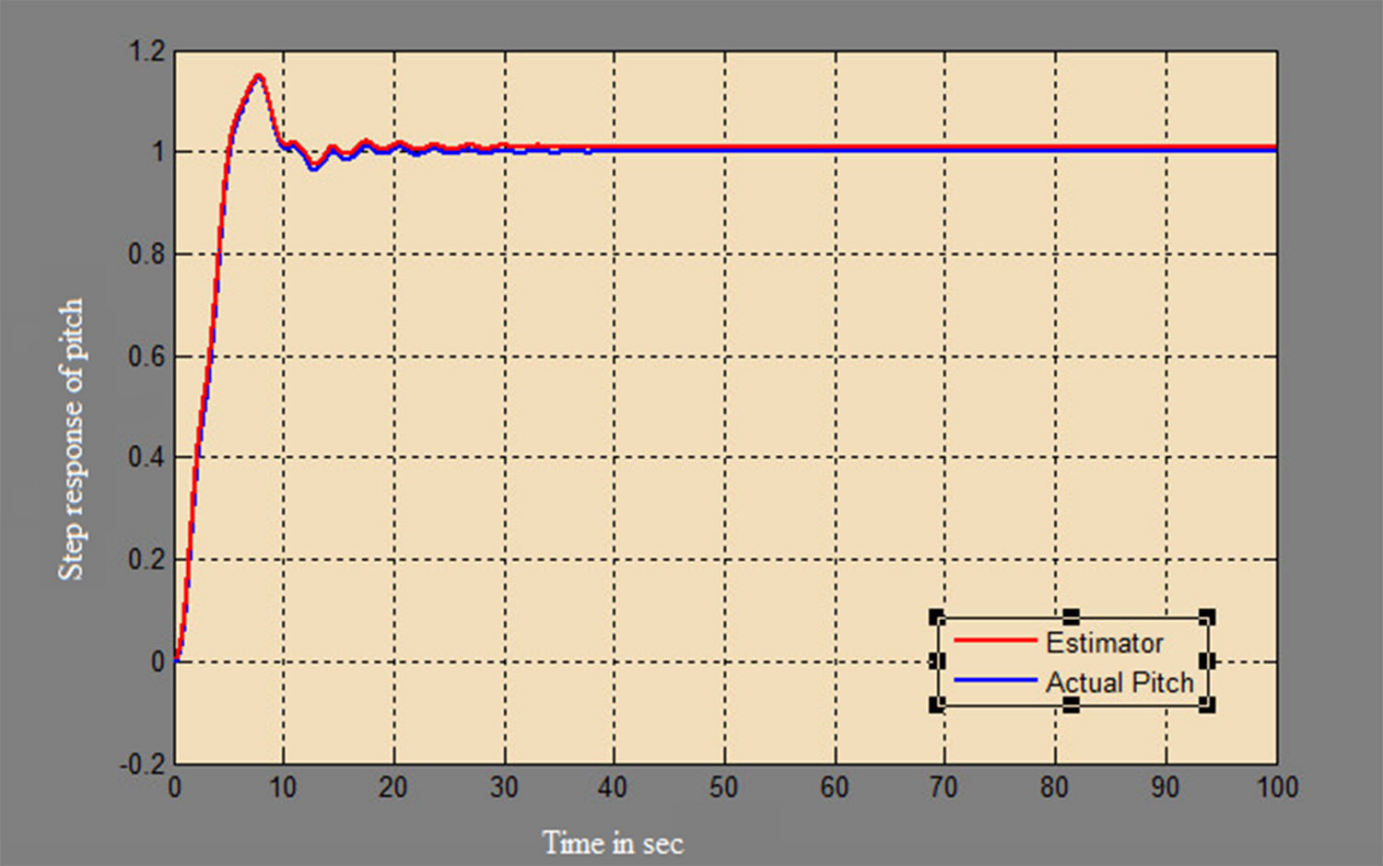
Estimation of pitch for a step input with Luenberger observer.

**Figure 10.**
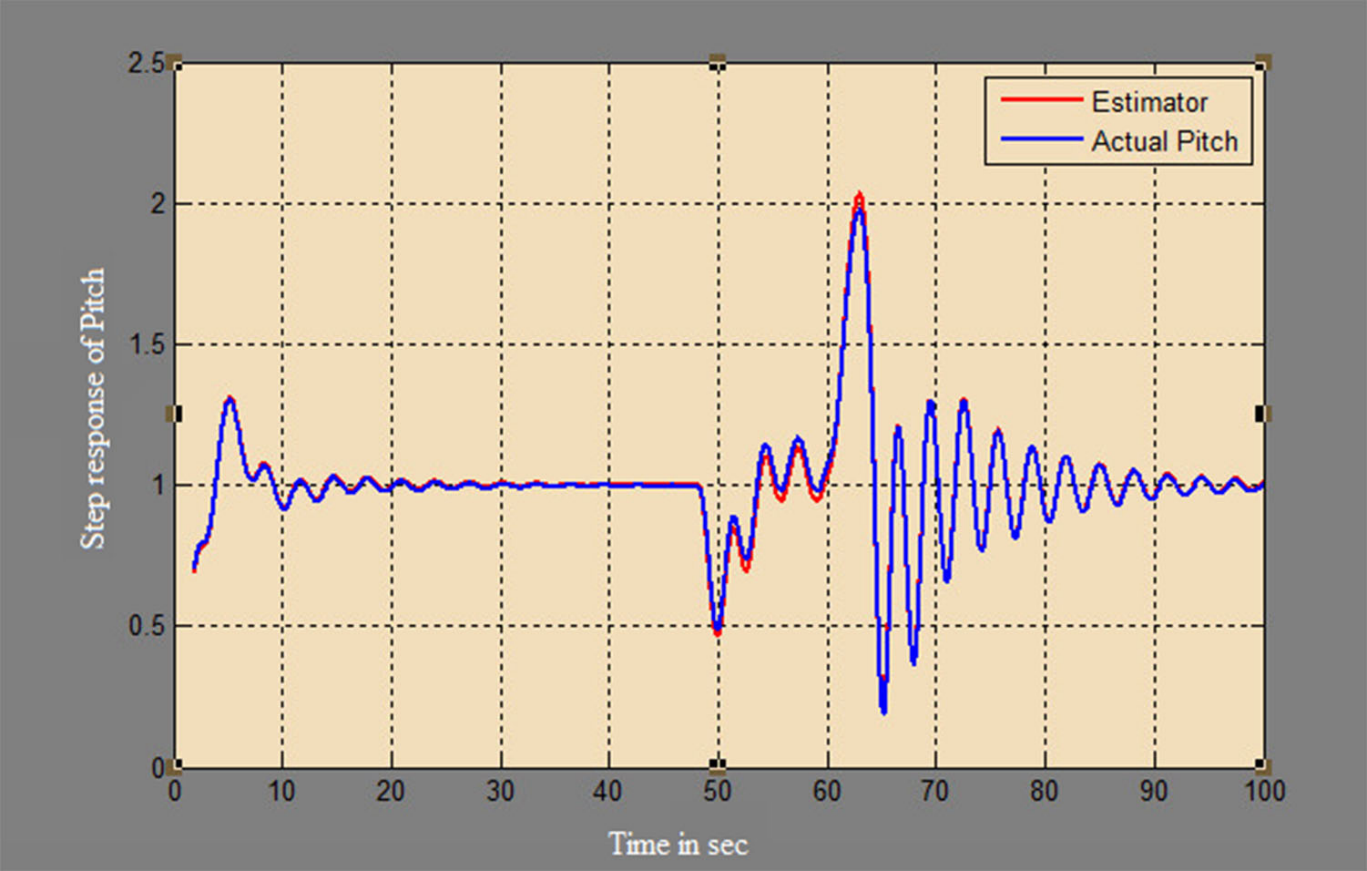
Estimation of pitch for a step input with disturbance with Luenberger observer.

**Figure 11.**
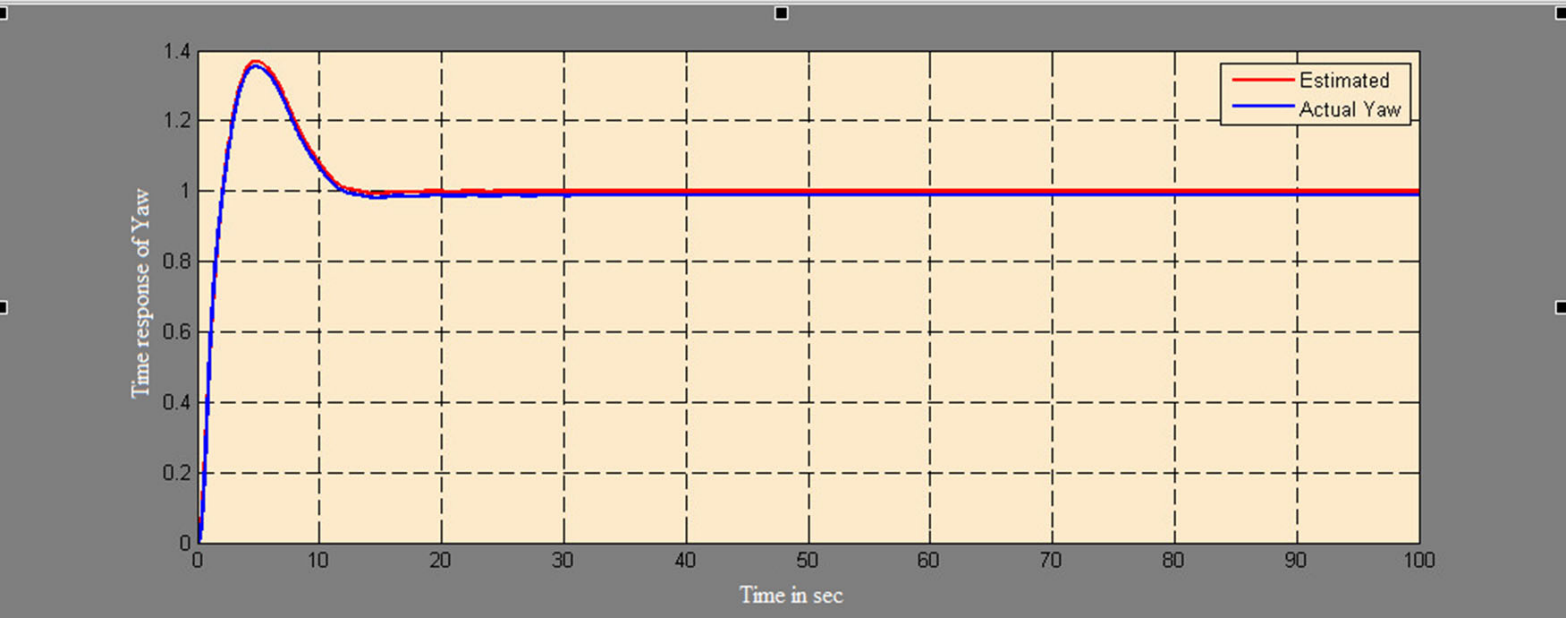
Estimation of yaw for a step input with Luenberger observer.

**Figure 12.**
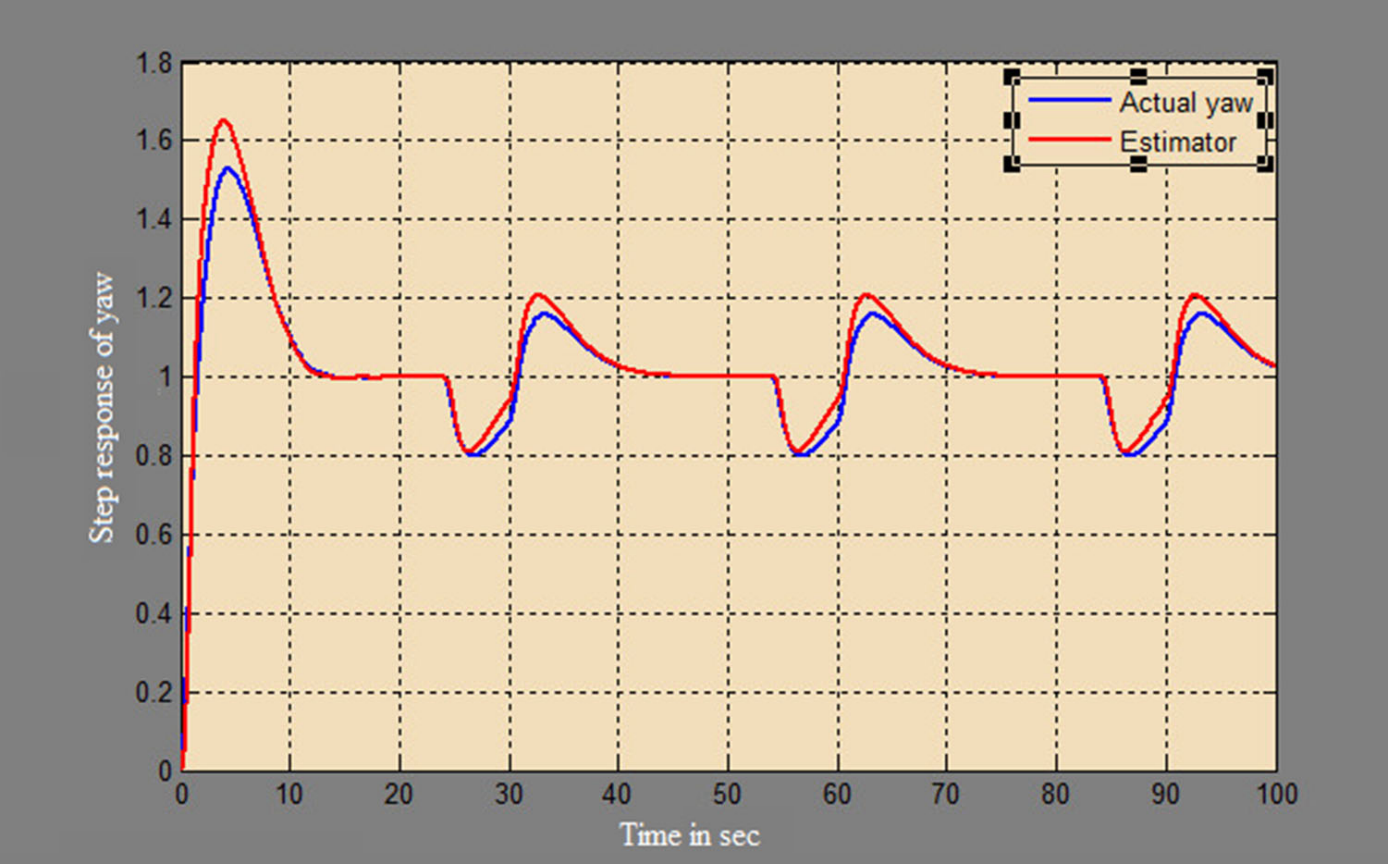
Estimation of yaw for a step input with disturbance with Luenberger observer.

**Figure 13.**
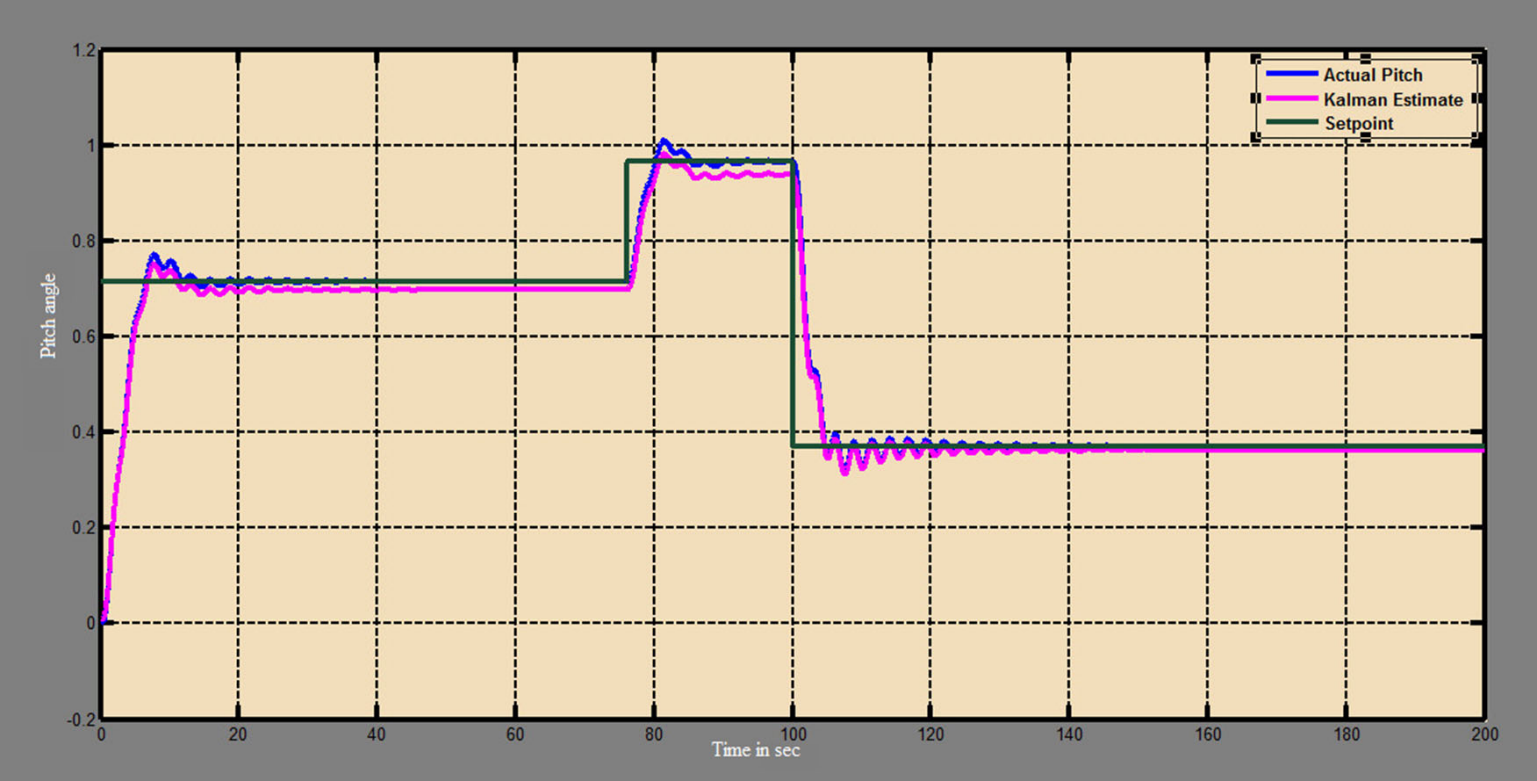
Estimation of pitch with Kalman filter.

**Figure 14.**
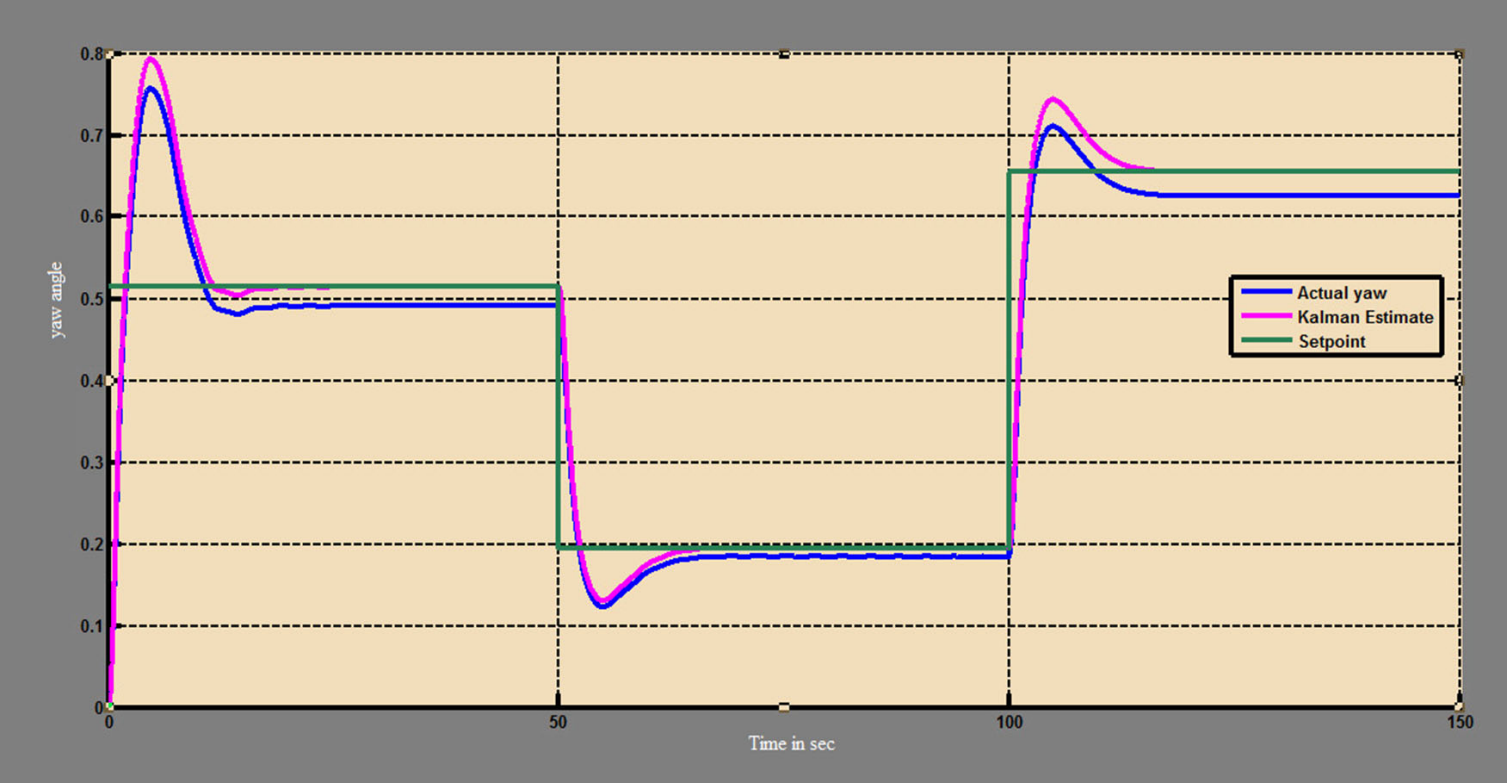
Estimation of yaw with Kalman filter.

**Figure 15.**
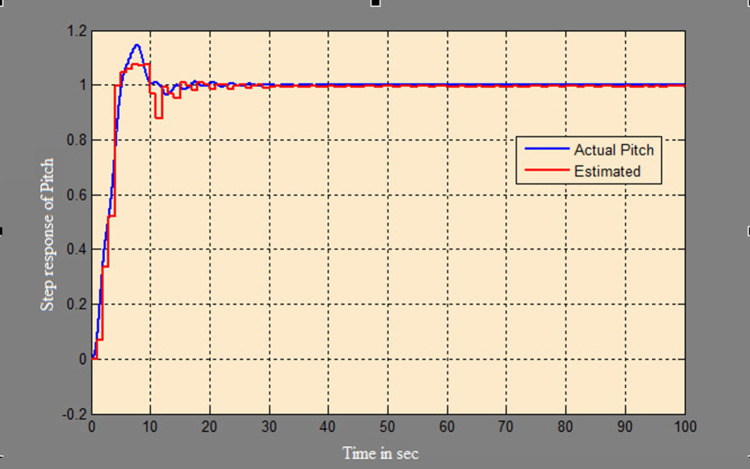
Estimation of pitch with neural network model.

**Figure 16.**
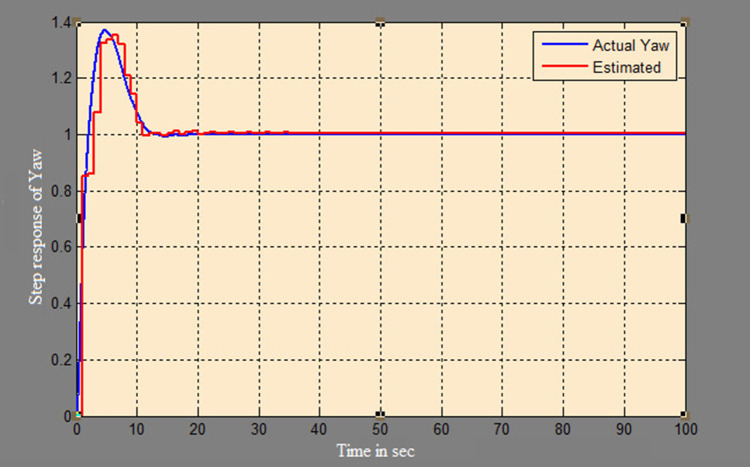
Estimation of yaw with neural network model.

**Figure 17.**
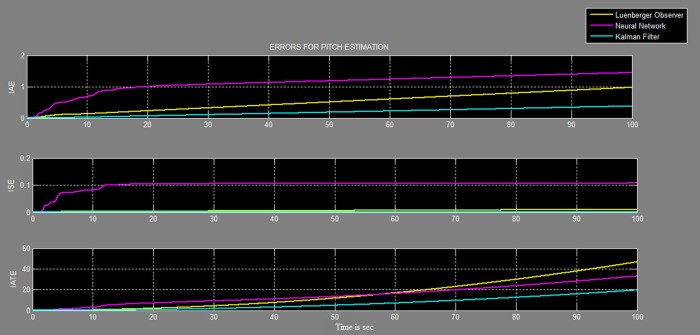
Comparative graph of errors in estimation of pitch with three different systems.

**Figure 18.**
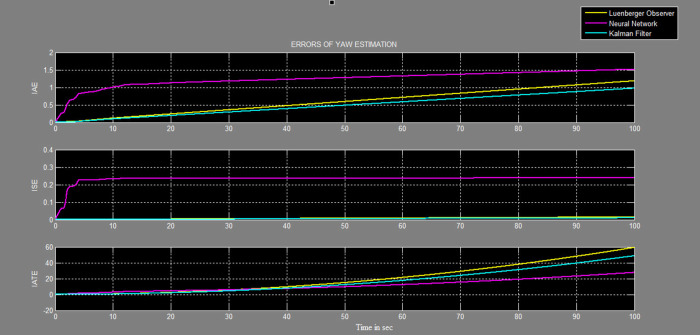
Comparative graphs of errors in estimation of yaw with three different systems.

A soft sensor for computing the yaw and pitch in TRMS using three different techniques, a neural network, Kalman filter, and a Luenberger observer, was designed. It was subjected to tests in real life. Tests were conducted by applying input and disturbance. The response of the proposed sensing system using the Luenberger observer is represented in Figures 9- 12. The output obtained for the Kalman filter is shown in Figures 13 and 14. Similarly, the output obtained from the neural network is shown in Figures 15 and 16. From the responses it was found that the proposed sensing technique was able to track the pitch and yaw positions accurately in a practical system. The performance measures IAE, ISE, and ITAE were used to quantitatively compare the outputs obtained from the Luenberger observer, Kalman filter, and neural network estimator. The results of these for the pitch and yaw measurement are shown in Figures 17 and 18, respectively. This method provided a way to classify the errors that occurred in different stages of the system operation and provided a means to judge the accuracy of estimation. The estimator having the highest number of errors was not accurate. The steady state of the system occurred at around time = 50 seconds, and the errors at that instant for all the estimators were tabulated for pitch and yaw measurements in Table 1. ^35^

**Table 1.** Performance errors of the three techniques: Kalman filter, Luenberger observer and neural network. Integral Absolute Error (IAE), Integral Square Error (ISE), Integral Time Absolute Error (ITAE).

	Pitch measure			Yaw measure		
	Kalman filter	Luenberger observer	Neural network	Kalman filter	Luenberger observer	Neural network
IAE	0.251	0.5086	1.18	0.5	0.69	1.28
ISE	0.004	0.005	0.11	0.038	0.04	0.22
ITAE	6.732	11.745	13.35	9.33	9	10.3

## Discussion and Conclusions

Twin rotor multi input systems are fundamental for any aerial system, analysis of any property on the TRMS makes it important when designing any system further. The TRMS contains a motor system along with a sensor system for testing. Sensors are an integral part of the TRMS for providing actual information to the controller. Failure of the sensors would lead to errors in control action and thus lead to system failure. An attempt was made in this work to design an observer-based system, which would function accurately even with sensor faults. The effectiveness of the Kalman filter algorithm for estimation of pitch and yaw angular positions was verified in real life on the TRMS with external disturbances. The estimate was updated at every sampling time to predict the yaw and pitch for angular positions for the given input and measurement data. The performance of the Kalman filter was compared with that of the neural network and Luenberger observer. From the results of IAE, ISE, and ITAE, the error was least when using the Kalman filter followed by the Luenberger observer and lastly, the neural network. Hence, the estimation by the Kalman filter was more accurate during both transient and steady states.

## Data Availability

### Extended Data

Open Science Framework: Extended data for ‘Design of a soft sensing technique for measuring pitch and yaw angular positions for a Twin Rotor MIMI System’, https://doi.org/10.17605/OSF.IO/52V9D. ^35^

This project contains the following extended data:

• Supplementary Video 1: Experimental model of a soft sensor design technique for estimation of pitch and yaw angular positions of a Twin Rotor MIMO System (TRMS)
• Supplementary Data 1: Simulink file used to carry out the real time experimentation of TRMS system.

Data are available under the terms of Creative Commons Zero “No rights reserved” data waiver (CC0 1.0 Public domain dedication).
