# Supplementary material for: Design of a soft sensing technique for measuring pitch and yaw angular positions for a Twin Rotor MIMO System
Source: F1000Res. 2021 Nov 25;10:342. Originally published 2021 May 5. [Version 2] doi: 10.12688/f1000research.51894.2 (PMC8772526; doi:10.12688/f1000research.51894.2)
Supplement: Peer review history

# Peer review: Reviewer response for version 2

**DOI:** 10.5256/f1000research.79455.r101128

**Published:** 2021-12-06

**Authors:** Sudhakar Kumarasamy

**Affiliations:** 1 Faculty of Mechanical and Automotive Engineering Technology, Universiti Malaysia Pahang, Pekan, Malaysia

The revised version is much improved in terms of presentation and content. It may be considered for indexing.

Is the work clearly and accurately presented and does it cite the current literature?

Yes

If applicable, is the statistical analysis and its interpretation appropriate?

Partly

Are all the source data underlying the results available to ensure full reproducibility?

Yes

Is the study design appropriate and is the work technically sound?

Yes

Are the conclusions drawn adequately supported by the results?

Partly

Are sufficient details of methods and analysis provided to allow replication by others?

Yes

Reviewer Expertise:

Solar energy, Energy modelling, Energy systems

I confirm that I have read this submission and believe that I have an appropriate level of expertise to confirm that it is of an acceptable scientific standard.

# Peer review: Reviewer response for version 2

**DOI:** 10.5256/f1000research.79455.r101129

**Published:** 2021-11-29

**Authors:** Abuabiah Mohammad

**Affiliations:** 1 Mechanical and Mechatronics Engineering Department, Faculty of Engineering & Information Technology, An-Najah National University, Nablus, Palestinian Territory

Thanks for the author's reply and for submitting the new version. Based on the new version, I confirm that I have no further comments to make.

Is the work clearly and accurately presented and does it cite the current literature?

Partly

If applicable, is the statistical analysis and its interpretation appropriate?

Not applicable

Are all the source data underlying the results available to ensure full reproducibility?

Yes

Is the study design appropriate and is the work technically sound?

Yes

Are the conclusions drawn adequately supported by the results?

Yes

Are sufficient details of methods and analysis provided to allow replication by others?

Partly

Reviewer Expertise:

Control, system identification, mechatronics systems, automotive

I confirm that I have read this submission and believe that I have an appropriate level of expertise to confirm that it is of an acceptable scientific standard.

# Peer review: Reviewer response for version 1

**DOI:** 10.5256/f1000research.55105.r92429

**Published:** 2021-09-21

**Authors:** Sudhakar Kumarasamy

**Affiliations:** 1 Faculty of Mechanical and Automotive Engineering Technology, Universiti Malaysia Pahang, Pekan, Malaysia

Nayak *et al.,* have carried out a study of soft sensing technique for measuring pitch and yaw angular positions for a Twin Rotor MIMO System. The research result is interesting. However some suggestions are provided to improve the quality and presentation of the study:

1. Please maintain consistency in the use of Terminologies. For instance , is it MIMO or MIMI?
2. Some sentences are difficult to read or incomplete (e.g. "Twin rotor multi input systems are fundamental for any aerial system, analysis of any property on the TRMS makes it important when designing any system further; It was subjected to tests in real life"). Sentence structure and rephrasing is needed.
3. Provide list of Abbreviations: TRMS, MIMO, MIMI IAE, ISE, and ITAE.
4. Instead of Comparative graphs, it may be mentioned as Comparative results.
5. Distinguish the various soft sensing technique in terms of the stochastic methods? Which among the studied are stochastic and deterministic?
6. The Luenberger observer *based system*, Kalman filter *algorithm and gain*, and neural network *model using Levenberg-Marquardt algorithm and gain*.
7. The various gain values and the noise covariance may be compared in a tabular format.
8. The transient, steady states, 1-DOF control and 2-DOF control may be explained a bit clearly in the methodology and results section.
9. Please provide the technical details of the TRMS containing the Twin Rotor motor system and the sensor system.
10. The neural network regression model results for both pitch and yaw control may be introduced under the results section.
11. What is the major contribution of this study? The soft sensing technique designed and tested for the senor motor by the authors is useful for other applications? Please elaborate on those aspects.
12. What are the major limitations and future scope of the study.
13. The conclusion lacks significant numbers to support the major outcome of the study.
14. List down the key conclusion on the various numerical values obtained from the study.

Is the work clearly and accurately presented and does it cite the current literature?

Yes

If applicable, is the statistical analysis and its interpretation appropriate?

Partly

Are all the source data underlying the results available to ensure full reproducibility?

Yes

Is the study design appropriate and is the work technically sound?

Yes

Are the conclusions drawn adequately supported by the results?

Partly

Are sufficient details of methods and analysis provided to allow replication by others?

Yes

Reviewer Expertise:

Solar energy, Energy modelling, Energy systems

I confirm that I have read this submission and believe that I have an appropriate level of expertise to confirm that it is of an acceptable scientific standard, however I have significant reservations, as outlined above.

# Response

**Published:** 2021-11-13

**Authors:** Santhosh Venkata

**Affiliations:** Manipal Academy of Higher Education, India

Reviewer 2:

 Comments: Nayak et al., have carried out a study of a soft sensing technique for measuring pitch and yaw angular positions for a Twin Rotor MIMO System. The research result is interesting. However, some suggestions are provided to improve the quality and presentation of the study:

- Please maintain consistency in the use of Terminologies. For instance, is it MIMO or MIMI?

 
**Response: **Sorry for the mistake, we have consistently used MIMO, in the revised article.

- Some sentences are difficult to read or incomplete (e.g. "Twin rotor multi-input systems are fundamental for any aerial system, analysis of any property on the TRMS makes it important when designing any system further; It was subjected to tests in real life"). Sentence structure and rephrasing are needed.

 
**Response: **Sorry for the mistake, the sentence is rephrased.

- Provide a list of Abbreviations: TRMS, MIMO, MIMI IAE, ISE, and ITAE.

 
**Response**: As suggested a table for abbreviation is provided.

- Instead of Comparative graphs, it may be mentioned as Comparative results.

** Response: **As suggested the comparative graphs is changed to comparative results

- Distinguish the various soft sensing technique in terms of the stochastic methods? Which among the studied are stochastic and deterministic?

 
**Response: **As suggested we have added details about the sensing technique.

- The Luenberger observer-based system, Kalman filter algorithm and gain, and neural network model using Levenberg-Marquardt algorithm and gain. The various gain values and the noise covariance may be compared in a tabular format.

** Response: **As suggested a comparative analysis of various system gains is provided in the revised version.

- The transient, steady states, 1-DOF control, and 2-DOF control may be explained a bit clearer in the methodology and results section.

 
**Response: **As suggested details are presented in the results section

- Please provide the technical details of the TRMS containing the Twin Rotor motor system and the sensor system.

 
**Response: **Thank you for the suggestion, we have included details of the TRMS.

- The neural network regression model results for both pitch and yaw control may be introduced under the results section.

 
**Response: **As suggested results of both pitch and yaw are moved to results sections.

- What is the major contribution of this study? The soft sensing technique designed and tested for the senor motor by the authors is useful for other applications? Please elaborate on those aspects.

** Response: **As suggested details are included in the conclusion.

- What are the major limitations and future scope of the study?

**Response: **As suggested details are included in the conclusions.

- The conclusion lacks significant numbers to support the major outcome of the study.

 
**Response: **As suggested, outcomes are included in the conclusion

- List down the key conclusion on the various numerical values obtained from the study.

**Response:** As suggested, numerical values are included in the conclusion.

# Peer review: Reviewer response for version 1

**DOI:** 10.5256/f1000research.55105.r92431

**Published:** 2021-09-16

**Authors:** Abuabiah Mohammad

**Affiliations:** 1 Mechanical and Mechatronics Engineering Department, Faculty of Engineering & Information Technology, An-Najah National University, Nablus, Palestinian Territory

**Contribution:**

 The paper addresses the problem of estimation pitch and yaw angular positions of a Twin Rotor MIMO System (TRMS) using the soft-sensor technique. In particular, the authors propose an estimation to calculate the value of pitch and yaw angular positions using Kalman filter, Luenberger observer and neural network. More specifically, measurements from optical sensors were used to measure fan blade rotations per minute (RPM), and then Kalman filter estimators were evaluated for various inputs and disturbances. Finally, different experimental results have been presented to compare Kalman filter estimation with those of the Luenberger observer and neural network. The results show that the Kelman filter has the least IAE, ISE and ITAE as compared to the two other mentioned methods.

**General Evaluation:**

 The submitted paper has some novelty in the field of soft sensing techniques using the Kelman filter approach for the Twin Rotor MIMO system. Although the paper suffers from a lack of deep analysis and comparison with other previous works, it is the reviewer opinion that the presented approach is of some interest since it provides an approach for estimating TRMS using Kalman filter in a comparison with Luenberger observer and neural network approach.

 All considered, it is my opinion that the paper deserves the merit to be considered for publication, provided that the comments reported below are properly taken into account by the authors.

**Comments:**

 The paper in general is organized. However, the authors are invited to take into account the following comments in order to improve the quality of the contribution.

 1) It is my opinion that the literature review is not satisfactory. Although the authors mentioned some of the previous methods related to the topic, they didn't provide a full literature overview of the proposed problem and more specifically to model estimation of the TRMS system. The authors are advised to add e.g., the work of A. Tastemirov *et al.* (2017) ^1^, R. Maiti *et al.* (2018) ^2^, S. Miah *et al.* (2019) ^3^, etc. and to provide a discussion on the comparison between such works and the proposed approach. 

 2) It is not clear on which basis the poles for the observer for both pitch and yaw were chosen to be at -1, -2, and -3. The authors are advised to add more details about the chosen values and how these can affect the accuracy of the obtained model.

 3) The matrices for the decoupled pitch and yaw models in Equations 3 and 4 are exactly the same; which is strange. The authors are advised to re-check again the values and discuss more the founded model. Moreover, the S _y_ matrix is not clear, there should be spaces between each column. 

 4) R and Q matrices computation was missing or not well discussed. The authors are advised to explain more the procedure they used to compute R and Q and how this leads to equation 10. 

 5) An explanation of Fig. 2 is missing. The authors are advised to comprehensively discuss the outline of this paper. Furthermore, it suggested removing any literature that is out of the scope of the provided topic (e.g., 23 - 26). 

 6) The authors stated the following "The estimator having the highest number of errors was not accurate" on page 11. However, it is not so clear and it is suggested to re-write it in a better way.

Is the work clearly and accurately presented and does it cite the current literature?

Partly

If applicable, is the statistical analysis and its interpretation appropriate?

Not applicable

Are all the source data underlying the results available to ensure full reproducibility?

Yes

Is the study design appropriate and is the work technically sound?

Yes

Are the conclusions drawn adequately supported by the results?

Yes

Are sufficient details of methods and analysis provided to allow replication by others?

Partly

Reviewer Expertise:

Control, system identification, mechatronics systems, automotive

I confirm that I have read this submission and believe that I have an appropriate level of expertise to confirm that it is of an acceptable scientific standard, however I have significant reservations, as outlined above.

## References

[1] : Complete dynamic model of the Twin Rotor MIMO System (TRMS) with experimental validation. *Control Engineering Practice* .2017;66: 10.1016/j.conengprac.2017.06.009 89-98 10.1016/j.conengprac.2017.06.009

[2] : PSO based parameter estimation and PID controller tuning for 2-DOF nonlinear twin rotor MIMO system. *International Journal of Automation and Control* .2018;12(4) : 10.1504/IJAAC.2018.095109 10.1504/IJAAC.2018.095109

[3] : Generalized Cascaded Control Technology for a Twin-Rotor MIMO System with State Estimation. *Journal of Control, Automation and Electrical Systems* .2019;30(2) : 10.1007/s40313-019-00448-z 170-180 10.1007/s40313-019-00448-z

# Response

**Published:** 2021-11-13

**Authors:** Santhosh Venkata

**Affiliations:** Manipal Academy of Higher Education, India

1) It is my opinion that the literature review is not satisfactory. Although the authors mentioned some of the previous methods related to the topic, they didn't provide a full literature overview of the proposed problem and more specifically to model estimation of the TRMS system. The authors are advised to add e.g., the work of A. Tastemirov et al. (2017)1, R. Maiti et al. (2018)2, S. Miah et al. (2019)3, etc. and to provide a discussion on the comparison between such works and the proposed approach.

**Response: **Thank you for the suggestion, we have included recent and relevant publications in the literature review.

 2) It is not clear on which basis the poles for the observer for both pitch and yaw were chosen to be at -1, -2, and -3. The authors are advised to add more details about the chosen values and how these can affect the accuracy of the obtained model.

**Response: **Any observer design aims to drive the error between the outputs of the plant and observer to zero. The error function is multiplied with the gain which enhances the convergence of the estimates to the true states. A high gain leads to fast convergence but too high causes a peaking phenomenon. By choosing the eigenvalues of the observer on the left half of the s-plane, the error dynamics can be controlled appropriately. For computation, poles are considered by multiplying by at least 10 times to the poles of the system.

 3) The matrices for the decoupled pitch and yaw models in Equations 3 and 4 are exactly the same; which is strange. The authors are advised to re-check again the values and discuss more the founded model. Moreover, the Sy matrix is not clear, there should be spaces between each column.

**Response: **The decoupled pitch model was found to be:

*F* *p* *=* *-* *1.4389* *-* *3.1862* 1.6706 *0.0803*-4.9874 -29.1821 *-* *0.0376* 0.0474 -5.5737;  *G* *p* *=* *1* *0* *0*  *S* *p* *=* *0.0166  0.4194  2.454* *;* *D* *=0*  (3).

 The matrices for the decoupled yaw model were found to be:

*F* *y* *=* *-* *1.38* *-* *1.6456* *-* *14.7611* *0.9244* *-* *2.5724* *-* *31.1124* *-* *0.0196* *0.3346* *-* *8.0476*;  *G* *y* *=* *1* *0* *0* 

*S* *y* *=* *0.001  0.0336  0.4065* *;  D=0* (4).

 Both the equations were rechecked. Sorry for the representation, F _y_ is a 3x3 matrix, G _y_ is 3x1 and S _y_ is a 1x3 matrix.

 4) R and Q matrices computation was missing or not well discussed. The authors are advised to explain more the procedure they used to compute R and Q and how this leads to equation 10.

**Response:** the noise covariance matrices R and Q are the most complex matrices to compute, therefore the idea is to start with an initial estimate of identity matrices for both R and Q and to change it based on the convergence between the actual and the estimated outputs.

 5) An explanation of Fig. 2 is missing. The authors are advised to comprehensively discuss the outline of this paper. Furthermore, it suggested removing any literature that is out of the scope of the provided topic (e.g., 23 - 26).

**Response: **Thank you for the suggestion, we have added an explanation for Figure 2 and removed the literature as suggested.

 6) The authors stated the following "The estimator having the highest number of errors was not accurate" on page 11. However, it is not so clear and it is suggested to re-write it in a better way.

**Response:** Sorry for that, we have revised the paragraph so, as to prove improvement to the work.
