# Supplementary material for: Design of a soft sensing technique for measuring pitch and yaw angular positions for a Twin Rotor MIMO System
Source: F1000Res. 2021 May 5;10:342. [Version 1] doi: 10.12688/f1000research.51894.1 (PMC8772526; doi:10.12688/f1000research.51894.1)
Supplement: Peer review history

# Response

**Published:** 2021-11-13

**Authors:** Santhosh Venkata

**Affiliations:** Manipal Academy of Higher Education, India

1) It is my opinion that the literature review is not satisfactory. Although the authors mentioned some of the previous methods related to the topic, they didn't provide a full literature overview of the proposed problem and more specifically to model estimation of the TRMS system. The authors are advised to add e.g., the work of A. Tastemirov et al. (2017)1, R. Maiti et al. (2018)2, S. Miah et al. (2019)3, etc. and to provide a discussion on the comparison between such works and the proposed approach.

**Response: **Thank you for the suggestion, we have included recent and relevant publications in the literature review.

 2) It is not clear on which basis the poles for the observer for both pitch and yaw were chosen to be at -1, -2, and -3. The authors are advised to add more details about the chosen values and how these can affect the accuracy of the obtained model.

**Response: **Any observer design aims to drive the error between the outputs of the plant and observer to zero. The error function is multiplied with the gain which enhances the convergence of the estimates to the true states. A high gain leads to fast convergence but too high causes a peaking phenomenon. By choosing the eigenvalues of the observer on the left half of the s-plane, the error dynamics can be controlled appropriately. For computation, poles are considered by multiplying by at least 10 times to the poles of the system.

 3) The matrices for the decoupled pitch and yaw models in Equations 3 and 4 are exactly the same; which is strange. The authors are advised to re-check again the values and discuss more the founded model. Moreover, the Sy matrix is not clear, there should be spaces between each column.

**Response: **The decoupled pitch model was found to be:

*F* *p* *=* *-* *1.4389* *-* *3.1862* 1.6706 *0.0803*-4.9874 -29.1821 *-* *0.0376* 0.0474 -5.5737;  *G* *p* *=* *1* *0* *0*  *S* *p* *=* *0.0166  0.4194  2.454* *;* *D* *=0*  (3).

 The matrices for the decoupled yaw model were found to be:

*F* *y* *=* *-* *1.38* *-* *1.6456* *-* *14.7611* *0.9244* *-* *2.5724* *-* *31.1124* *-* *0.0196* *0.3346* *-* *8.0476*;  *G* *y* *=* *1* *0* *0* 

*S* *y* *=* *0.001  0.0336  0.4065* *;  D=0*  (4).

 Both the equations were rechecked. Sorry for the representation, F _y_ is a 3x3 matrix, G _y_ is 3x1 and S _y_ is a 1x3 matrix.

 4) R and Q matrices computation was missing or not well discussed. The authors are advised to explain more the procedure they used to compute R and Q and how this leads to equation 10.

**Response:** the noise covariance matrices R and Q are the most complex matrices to compute, therefore the idea is to start with an initial estimate of identity matrices for both R and Q and to change it based on the convergence between the actual and the estimated outputs.

 5) An explanation of Fig. 2 is missing. The authors are advised to comprehensively discuss the outline of this paper. Furthermore, it suggested removing any literature that is out of the scope of the provided topic (e.g., 23 - 26).

**Response: **Thank you for the suggestion, we have added an explanation for Figure 2 and removed the literature as suggested.

 6) The authors stated the following "The estimator having the highest number of errors was not accurate" on page 11. However, it is not so clear and it is suggested to re-write it in a better way.

**Response:** Sorry for that, we have revised the paragraph so, as to prove improvement to the work.
